# Adaptation of an Invasive Pest to Novel Environments: Life History Traits of *Drosophila suzukii* in Coastal and Mainland Areas of Greece during Overwintering

**DOI:** 10.3390/biology10080727

**Published:** 2021-07-29

**Authors:** Stella A. Papanastasiou, Vasilis G. Rodovitis, Eleni Verykouki, Evmorfia P. Bataka, Nikos T. Papadopoulos

**Affiliations:** Department of Agriculture, Crop Production and Rural Environment, University of Thessaly, Fytokou St., 38446 Volos, Greece; spapanast@uth.gr (S.A.P.); rodoviti@uth.gr (V.G.R.); everykouki@uth.gr (E.V.); bataka@uth.gr (E.P.B.)

**Keywords:** spotted wing drosophila, longevity, offspring, seasonal phenotypes, reproduction, fertility, winter morph, Drosophilidae

## Abstract

**Simple Summary:**

*Drosophila suzukii*, also known as the spotted wing Drosophila, is a notorious pest of several high-value fruits including strawberries and sweet cherries. Adult *D. suzukii* flies exhibit two morphs: summer morphs (SM) and winter morphs (WM). The two seasonal phenotypes help this pest to perform better in temperate climates. WM have a darker cuticle and larger wings compared to SM, while WM females experience reproductive dormancy. We estimated the lifespan, the reproductive status of females and the number of produced offspring for WM and SM exposed to mild and cold winter field conditions, prevailing in two different geographic areas (coastal and mainland). Overall, WM exhibited a longer lifespan than SM and this difference was more pronounced for adults kept in the cold mainland area. The majority of SM females produced offspring during overwintering in the mild coastal area, but only a few SM were reproductively active in the cold mainland area. Some WM females produced progeny during overwintering in the mild conditions of the coastal area, but all WM females were in reproductive arrest in the mainland area. Overwintering females in the coastal area had a shorter lifespan and produced more progeny than those kept in the mainland area. High survival rates of WM provide indications of the successful performance of this phenotype in the adverse conditions of the cold climates. Additionally, the continuous reproductive activity of SM females and the onset of progeny production by WM females during overwintering in the coastal area indicate that the insect remains reproductively active throughout the year in areas with mild climatic conditions. Our findings support the successful adaptation of *D. suzukii* in both areas tested and can be used for the development of area-specific population models, based on the prevailing climatic conditions.

**Abstract:**

*Drosophila suzukii* is a polyphagous pest of small and soft fruit, originating from Asia, which has spread and established in Europe and the USA. Adults exhibit seasonal phenotypes, i.e., summer morphs (SM) and winter morphs (WM) to cope with fluctuating environmental conditions. WM have a darker cuticle and larger wings compared to SM, while WM females experience reproductive dormancy. We studied the life history traits (lifespan, female reproductive status and number of produced offspring) of WM and SM that were exposed to winter field conditions of a coastal and a mainland agricultural area, with mild and cold winter climates, respectively. Mated adults of each phenotype were individually placed in vials bearing nutritional/oviposition substrate, and transferred to the field from November 2019 to May 2020, when the death of the last individual was recorded. Almost all SM females (90%) and no WM female carried mature ovarioles before being transferred to the field. WM exhibited a longer lifespan than SM adjusting for location and sex. Differences in survival between the two phenotypes were more pronounced for adults kept in the mainland area. The majority of SM females produced offspring during overwintering in the mild coastal area, but only a few SM were reproductively active in the cold mainland area. Some WM females produced progeny during overwintering in the mild conditions of the coastal area, but all WM females were in reproductive arrest in the mainland area. Overwintering females in the coastal area had a shorter lifespan and produced more progeny than those kept in the mainland area. High survival rates of WM provide indications of the successful performance of this phenotype in the adverse conditions of the cold climates. Additionally, the continuous reproductive activity of SM females and the onset of progeny production by WM females during overwintering in the coastal area indicate that the insect remains reproductively active throughout the year in areas with mild climatic conditions. Our findings support the successful adaptation of *D. suzukii* in both areas tested and can be used for the development of area-specific population models, based on the prevailing climatic conditions.

## 1. Introduction

*Drosophila suzukii* Matsumura (Diptera: Drosophilidae), also known as the Spotted Wing Drosophila (SWD), is a notorious pest of soft and small fruits such as berries, strawberries and sweet cherries [1,2,3]. It can also infest a wide range of wild plants, by producing several generations per year (multivoltine) and by expressing an extremely high reproductive potential [4]. Characterized by an increased invasive ability, the native to southeastern Asia *D. suzukii* has spread in most regions of Asia, and invaded Europe and the Americas during the last thirteen years [5]. Several European Mediterranean countries such as Spain, France and Italy reported the first detections during 2008–2009 [6]. By 2016, the presence of *D. suzukii* was confirmed in almost all of Europe, including Greece, as reviewed in [7,8,9]. Due to the relatively recent invasion and establishment of this pest in new regions, not much is known regarding the adaptability of populations in the newly invaded environments. Therefore, there is a growing need for investigating the effects of environmental abiotic factors on the life history traits of *D. suzukii* populations. Accumulating this type of information for different geographic areas will contribute in designing successful management strategies, applicable in fruit producing areas where the pest is present.

Phenotypic plasticity, the ability of a given genotype to produce different phenotypes in relation to varying environmental stimuli and seasonal changes, is a common characteristic of several temperate insect species [10]. Seasonal morphs in insects can manifest with temporal variation in color and body size, in a way that alternative phenotypes are produced through seasons to match with the conditions where their highest fitness (survival and/or reproduction) is expressed [11]. Cuticular melanization, increased body size and wing elongation are some of the typical adaptations of seasonal polymorphism, also manifested in overwintering *D. suzukii* adults, known as Winter Morphs (WM) [12]. The development of immatures at relatively low temperatures and a short photoperiod results in the emergence of WM adults, while immature development at higher temperatures and a long photoperiod results in the emergence of Summer Morphs (SM). The two seasonal morphs differ in metabolic rates and gene expressions indicating variations in cold tolerance and reproductive potential [12]. WM flies are better adapted to cold winter conditions where higher survival rates are recorded, while SM live longer in spring-like conditions [13,14,15,16,17]. Overwintering WM females have lower reproductive potential than SM but are the first to reach reproductive maturity and deposit their eggs early in the spring [18,19]. Depending on the geographic areas of the Northern hemisphere, WM can be trapped from September to June. On the other hand, SM can be active throughout the year in warmer areas, or from late spring until mid-autumn in colder areas, with both cases leading to co-occurrence of the two phenotypes during autumn and spring [20,21]. Understanding the adaptation of *D. suzukii* to cold and its effects on life history traits during overwintering is important for predicting population dynamics in temperate areas.

Life history traits of *D. suzukii* have been extensively studied under laboratory conditions [4,22,23,24], providing basal information regarding the adaptability of populations in novel environments [5,25]. Simulations of summer and winter conditions in the laboratory have shown that immature development, adult reproductive maturation, survival and fecundity of several temperate insects, including *D. suzukii,* are greatly affected by abiotic factors such as temperature and relative humidity (RH) [4,14,23,26,27]. Warm temperatures and high RH induce faster larval development, increased oviposition and survival rates in SM flies [4,24,26]. In addition, acclimated SM increase their survival rates and even outlive their equivalent WM under mild winter and spring-like conditions, indicating successful overwintering of SM in warmer areas [14,28]. Differences in survival rates under laboratory conditions, have also been recorded between males and females of several *D. suzukii* populations [29], as well as between the two phenotypes of the same sex [14]. Males appear globally more cold tolerant and less heat tolerant than females [13], while the mating status of both sexes does not affect their survival rates during overwintering [14,30]. However, a clear trend in lifespan differences between males and females of the two phenotypes has not been recorded yet, possibly indicating different survival responses of sex/phenotype combinations under the same temperature conditions [12,13]. Demographic experiments under laboratory conditions provide limited information because abiotic factors are tested under narrow ranges and restricted combinations to avoid confounding results. Therefore, it is usually difficult to interpret these findings and make safe conclusions regarding demographic responses in the field, where environmental factors are multi-dimensional and show great variability. Due to the high dispersion and establishment of *D. suzukii* in new environments, the susceptibility of overwintering adults to the conditions of temperate areas needs to be further assessed.

Under optimum temperature (20–25 °C), *D. suzukii* females reach reproductive maturation and mate within a few hours, initiate egg-laying one to four days post-eclosion, and reach their reproductive peak after 20 days [4,5,24,31]. Subsequently, fecundity rates start to gradually decline, although, unlike other drosophilids, *D. suzukii* females continue ovipositing until advanced ages (>40 days) [4,5]. Larval development during autumn and winter in the field or under low temperatures and a short photoperiod during artificial rearing results in the eclosion of reproductively halted *D. suzukii* females carrying dormant ovaries with no developed eggs [18,32]. However, it is not yet clear whether these females entail a true reproductive diapause [31,33]. The intensity of reproductive arrest in WM females may vary among areas with different latitudes and altitudes leading to diverse onsets of egg production and fecundity peaks during spring and summer [34]. Previous studies show that both the reproductive maturation and the oviposition onset of WM females is achieved rather quickly after a short exposure to high or spring-like temperatures [17,31]. Nevertheless, no information is available regarding the course of progeny production by reproductively active SM females experiencing autumn and winter environmental conditions. Is reproduction abruptly stopped or gradually decreased as temperature drops and photophase declines? How do SM females respond to seasonal changes in terms of survival? It is known that SM females have higher reproductive potential than WM females under optimum temperatures (20–25 °C) [14], but the survival and reproduction rates of the two phenotypes have not been assessed under winter field conditions.

Despite the vast information regarding survival and fecundity of *D. suzukii* under different laboratory conditions, limited information exists regarding the life history traits of this pest in field conditions. Field cage experiments in San Joaquin Valley of California (where freezing events were recorded in December and mean daily temperature was mostly below 10 °C during winter 2013) showed successful overwintering at the adult stage in this temperate area, coupled with low progeny production [35]. Although earlier studies showed that offspring production stops at temperatures below 10 °C [4,28], the winter temperature in San Joaquin did not induce a pause in reproductive activities. Overwintering adults were active and even engaged in copulations during the warmest period of several days. While no information regarding morph status was given in this study, overwintering females were, interestingly, able to produce viable offspring, indicating that the fluctuating winter temperatures of this area were not sufficient to cause reproductive arrest. Combined overwintering patterns of the two seasonal morphs of *D. suzukii* have not been explored yet in field conditions and would bring important information regarding the survival and reproduction dynamics of this pest in temperate areas.

The aim of the present study was to determine the overwintering capacity of the two seasonal phenotypes of *D. suzukii* under different environmental conditions. We tested the hypothesis that WM perform better in winter conditions than SM in both a mild coastal and a cold mainland area, and exhibit a longer lifespan than SM. Our key questions include the following: (a) Is successful overwintering achieved by both phenotypes under cold and mild winter climates? (b) What is the effect of a mild and a cold winter on progeny production by the two phenotypes? (c) Does one morph confer fitness benefits compared to the other? Our study provides answers by recording the survival and offspring production of *D. suzukii* SM and WM when exposed to mild and cold winter conditions of two distinct agricultural areas of central and northern Greece, respectively.

## 2. Materials and Methods

### 2.1. Rearing of Flies

Since 2018, a small-scale rearing of *D. suzukii* has been maintained under constant conditions (23 ± 2 °C, 55 ± 5% R.H., L14:D10 photoperiod) at the Laboratory of Entomology and Agricultural Zoology, University of Thessaly. The colony started with approximately 200 Summer Morph (SM) flies reared from infested sweet cherries, which were collected during July in the sweet cherry orchards of Northern Greece (Pella). Glass vials (Ø50 mm, 200 mL) furnished with a 2 cm layer of standard cornmeal diet (~30 mL) were used for the maintenance of the colony (approx. 30 females and 30 males per vial). Glass vials were sealed with a piece of cotton ball which prohibited adult escape and provided sufficient ventilation.

We used a cornmeal diet with sugar and protein at a 4:1 ratio, which stood both as nutritional and oviposition substrate. This contained agar, sucrose, cornmeal and baker’s yeast. For the preparation of a final volume of 1400 mL, the above ingredients (8, 70, 80, 17 gr, respectively) were cooked in 1330 mL water, at a gentle boil for approximately 10 min, until the homogenization of the mixture. A preservative (20 mL of 10% Nipagin in ethylic alcohol) was added once the mixture chilled (~60 °C). The diet was divided between the vials by covering their base to approximately 2 cm of height. Vials with fresh nutritional substrates were kept at room temperature (~20 °C) for 24 h before use, to achieve a solid phase.

### 2.2. Production of Summer and Winter Morphs

Seven to ten-day-old parental flies of F_4_ generation, obtained from the colony, were kept in glass vials (approx. 30 females and 30 males) for 48 h, allowing gravid females to oviposit in the fresh cornmeal substrate. Subsequently, adult flies were removed and the vials were transferred to climate chambers where larval development, pupation and adult emergence of either SM or Winter Morphs (WM) took place. For the production of SM, glass vials were transferred to SM chambers at 23 ± 2 °C, 55 ± 5% R.H. and a photoperiod of L14:D10. For the production of WM, glass vials were transferred to WM chambers at 13 ± 2 °C, 55 ± 5% R.H. and a photoperiod of L10:D14. Adult emergence of SM and WM was recorded at approximately 8 to 10 days and 30 to 35 days after oviposition, respectively [17].

### 2.3. Mating and Assessment of Ovarian Development of SM and WM Females

Before being transferred to the field, all vials containing SM and WM were kept for 72 h at the same acclimated chambers where the production of each phenotype took place. During this period SM and WM had reached reproductive maturity and had the opportunity to engage in copulations [14,30]. Subsequently, two samples of twenty randomly taken SM and WM females, were dissected to determine their ovarian development. Dissections were made using fine forceps and a phosphate-buffered saline solution under a binocular stereomicroscope. Ovarian development was classified into three stages: (i) undeveloped ovaries with no visible ovarioles, (ii) developing ovaries with small ovarioles visible and (iii) mature ovarioles with visible eggs [18,20,33].

### 2.4. Climatic Conditions Prevailing in the Mainland and the Coastal Area

Climatic data of the two agricultural areas Volos (coastal area with mild winter climate) and Naousa (mainland area with cold winter climate), where we performed our field trials, were obtained from two weather stations located in the respective areas (http://meteosearch.meteo.gr/ (accessed on 15 July 2020)). Maximum, mean and minimum daily temperature, as well as maximum and minimum relative humidity were recorded from November 2019 to May 2020, during the flies’ exposure to field conditions.

### 2.5. Exposure of Flies to Field Conditions: Recording Survival Rates and Progeny Production during Overwintering

The fourth day after adult emergence, SM and WM were sexed and individually placed in plastic, cylindrical vials (Ø30 mm, 50 mL), along with nutritional/oviposition substrate. The vials were sealed with a screwcap lid bearing a Ø25 mm opening for ventilation, covered with fine organdie cloth. Due to sufficient ventilation, temperature and humidity levels inside the vials matched those of ambient conditions. Fifty SM males and fifty SM females, as well as 25 WM males and equal number of WM females were subjected to the autumn and winter environmental conditions of two cherry producing areas of Greece: Volos (Magnisia County, central Greece, coastal area with mild winter) and Naousa (Pella County, northern Greece, mainland area with cold winter) (Table 1).

In total, 300 adults were observed daily from 1 November 2019 to 31 May 2020 for locomotion activity to determine whether they were alive. Inactive adults were carefully poked with a soft brush to confirm death. Offspring production by SM and WM females was also recorded through pupae formation within the nutritional substrate, which served also as a medium for larval development. The number of pupae was recorded daily and newly formed pupae were removed from the vial using soft forceps. The time-periods before, during and after recording pupae in the nutritional substrate were defined as pre-reproductive, reproductive and post-reproductive periods, respectively. Vials with fresh substrate were provided to adults when microbial degradation and/or dehydration of the medium was observed.

### 2.6. Statistical Analysis

We used the Cox proportional hazards model to assess possible effects of the area (coastal, mainland), the phenotype (SM, WM) and the sex (males, females) on adult mortality rates. This model is frequently used in demographic research to test the association between several predictors and time-to-event (i.e., death, conclusion of a time period). We also used the Kaplan–Meier estimate to depict survival patterns of the two phenotypes. The Cox proportional hazards model was also used to test the effects of the area and the phenotype on the duration of pre-reproduction, reproduction and post-reproduction period of females.

Using the Chi-square test, we compared the percentage of females bearing undeveloped and developing ovaries and mature ovarioles. Additionally, a binary logistic regression was performed to ascertain the effects of area and phenotype on the likelihood that females produce offspring. A multivariate Generalised Estimating Equation (GEE) Poisson regression model was used to estimate the number of progeny produced by SM and WM females that were overwintering in the two areas (coastal: Volos and mainland: Naousa) in relation to time since first exposure to field conditions, including the mean daily temperature per location as a covariate. Parameter estimates were presented as Incidence Rate Ratios (IRR) with 95% confidence intervals (CI), which is the ratio of the number of progeny in a group of interest to the number of progeny of the group used as reference. IRRs greater than 1 indicate more progeny for the group of interest while IRRs lower than 1 indicate more progeny for the reference group. Differences in climatic data (maximum, mean and minimum daily temperature, maximum and minimum daily relative humidity) between the two areas (coastal: Volos and mainland: Naousa), from November 2019 until May 2020, were assessed using the Prais–Winsten regression which takes into account AR(1) serial correlation of the errors in a linear regression model [36].

Data analyses were conducted with the statistical software SPSS 26.0 (SPSS Inc., Chicago, IL, USA) and with R v4.0.0 (R Core Team 2013, R Foundation of Statistical Computing, Vienna, Austria) in RStudio v1.1.453 (RStudio 2012, R Foundation of Statistical Computing, Vienna, Austria). GEE analysis was conducted using the package “geepack” [37,38,39] while the “prais” package was used for the Prais–Winsten regression [40], in R.

## 3. Results

### 3.1. Climatic Conditions Prevailing in the Mainland and the Coastal Area

**Figure 1:**
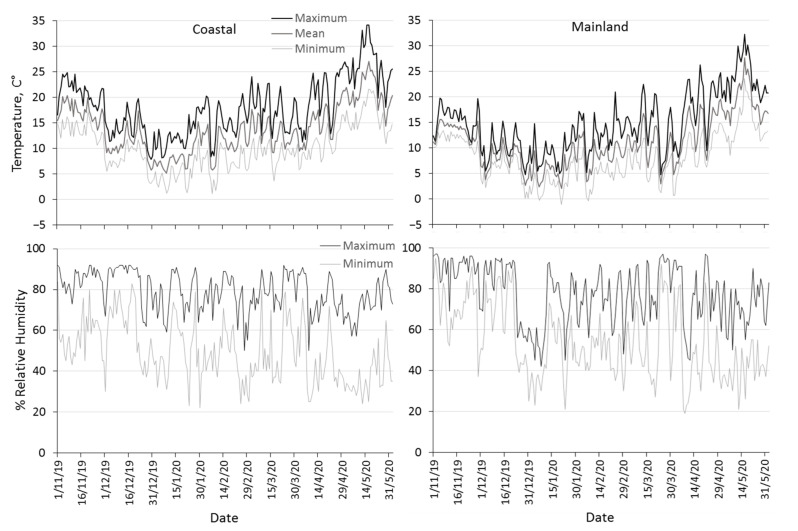
Seasonal patterns of maximum, mean and minimum daily temperature, maximum and minimum daily relative humidity prevailing in the coastal area Volos (**left column**) and the mainland area Naousa (**right column**) from 1 November 2019 to 31 May 2020.

Mean and maximum daily temperature were significantly lower in the mainland than in the coastal area (Table 2). The mean daily temperature in the coastal area ranged from 13.5 °C to 20.4 °C in November, from 5.2 °C to 17.4 °C during winter and from 9.2 °C to 27.1 °C in spring (Figure 1). The mean daily temperature in the mainland area was approximately 3–5 °C lower than that prevailing in the coastal area, and ranged from 10.5 °C to 15.5 °C in November, from 2 °C to 13.2 °C during winter and from 2.9 °C to 27.6 °C during spring. The mean daily temperature during winter in the coastal area was constantly above 5 °C but several cases of lower temperatures than 5 °C were recorded during winter in the mainland area. The absolute minimum temperature never dropped below zero in the coastal area, but freezing events were recorded during three days in the mainland area. The mainland area was characterized by higher relative humidity with greater fluctuations than the range of relative humidity in the coastal area, but no significant differences in humidity levels between the two areas were observed (Table 2, Figure 1).

### 3.2. Survival Rates of Overwintering Adults

**Figure 2:**
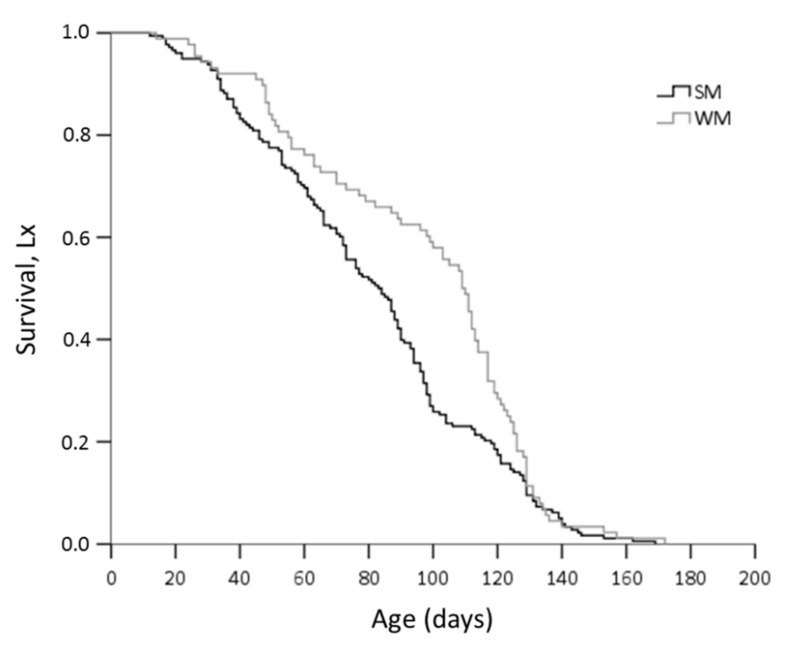
Kaplan–Meier survival estimates for *D. suzukii* SM and WM, adjusting for sex and the area where overwintering took place.

**Figure 3:**
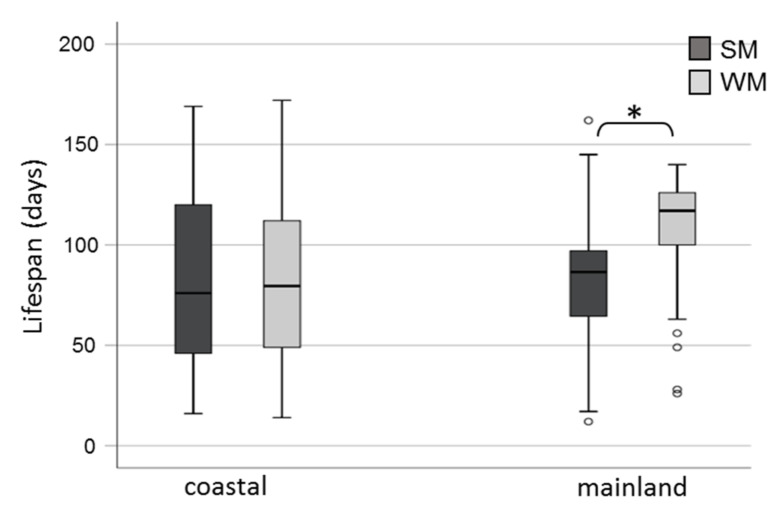
Lifespan (boxplots) of *D. suzukii* SM and WM exposed to field conditions in a coastal area of central Greece (Volos) and a mainland area of northern Greece (Naousa) from 1 November 2019 until the death of all individuals in May 2020. Values within the chart below the asterisk differ significantly (*p* < 0.05). Circles depict outliers (numerically distant values from the rest of the observations).

**Figure 4:**
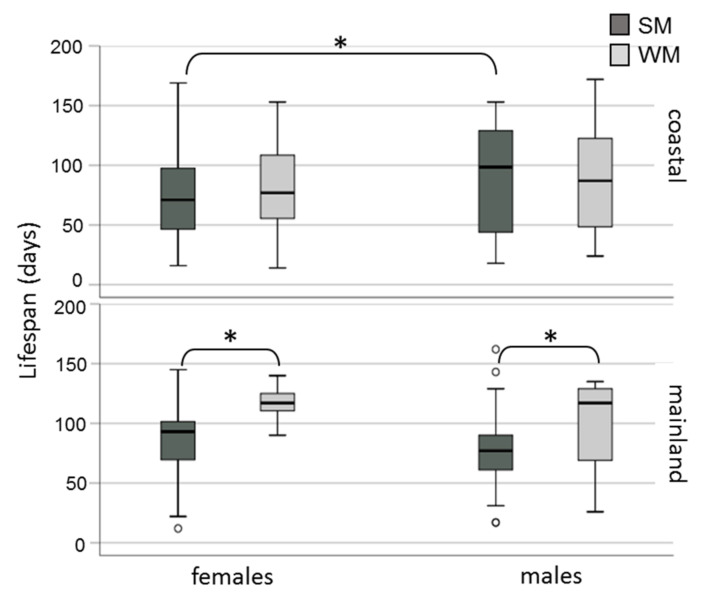
Lifespan (boxplots) of SM and WM of *D. suzukii* females and males exposed in field conditions in a coastal area of central Greece (Volos) and a mainland area of northern Greece (Naousa) from 1 November 2019 until the death of all individuals in May 2020. Values within the chart below the asterisk differ significantly (*p* < 0.05). Circles depict outliers (numerically distant values from the rest of the observations).

Winter morphs (WM) outlived summer morphs (SM) adjusting for area and sex (Wald test *t* = 6.192, df = 1, *p* = 0.013) (Figure 2). No significant differences in survival rates were observed either between adults exposed in the two areas (Wald test *t* = 0.004, df = 1, *p* = 0.952) or between males and females (Wald test *t* = 2.463, df = 1, *p* = 0.117). When the effect of the phenotype was tested against survival for each area separately, we found that WM flies lived longer than SM only when they were exposed to mainland field conditions (Wald test *t* = 14.134, df = 1, *p* = 0.0001) and not when they were kept in the coastal area (Wald test *t* = 0.06, df = 1, *p* = 0.806) (Figure 3). Overwintering females lived longer in the cold mainland than in the mild coastal area, adjusting for morph (Wald test *t* = 9.047, df = 1, *p* = 0.003). No significant differences in lifespan were detected between males overwintering in the two areas (Wald test *t* = 2.199, df = 1, *p* = 0.138). The longest-lived WM was a male exposed to the coastal area with recorded lifespan of 172 days. The longest lifespan recorded for SM was 169 days, by a female fly kept in the coastal area. In the coastal area, SM males outlived SM females (Wald test *t* = 6.421, df = 1, *p* = 0.011) (Figure 4).

### 3.3. Reproductive Rates of Overwintering Females

**Figure 5:**
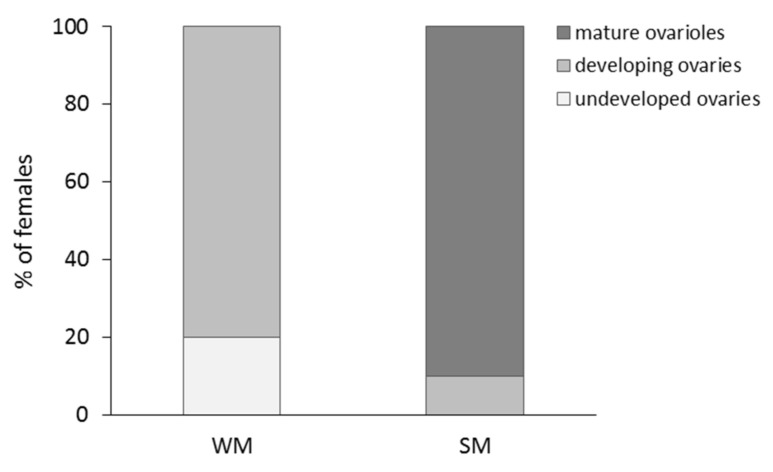
Percentage of WM and SM females with different levels of reproductive maturity. *Drosophila suzukii* females were dissected 72 h after eclosion, and before all individuals were subjected to the field conditions of the coastal (mild winter) and the mainland (cold winter) area.

**Figure 6:**
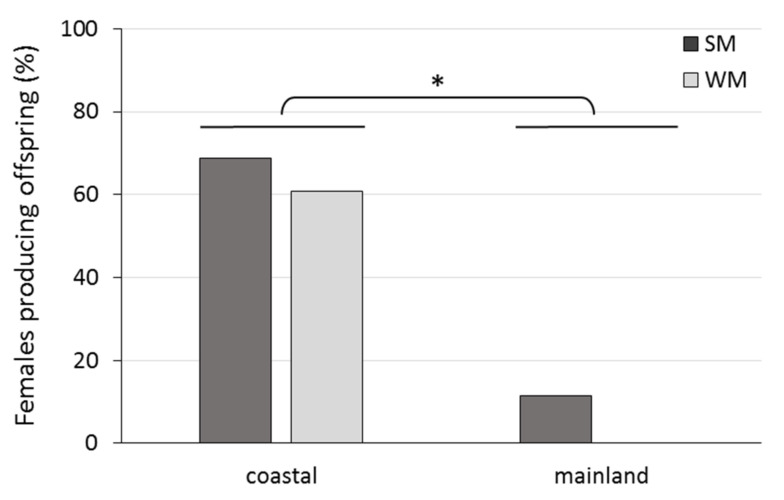
Percentage of SM and WM females that produced offspring during their exposure to field conditions of a coastal (Volos) and a mainland (Naousa) area from 1 November 2019 until the death of all individuals in May 2020. Values within the chart below the asterisk differ significantly (*p* < 0.001).

The percentage of WM and SM females bearing undeveloped and developing ovaries and mature ovarioles is given in Figure 5. No WM female was found to possess mature ovarioles. The majority of WM females (80%) had developing ovaries, and the rest (20%) undeveloped ovaries. On the other hand, only 10% of SM females had developing ovaries and 90% of SM females had mature ovarioles with visible eggs (Chi-square test = 32.889, df = 2, *p* < 0.001). Females were 22.9 times more likely to produce offspring when they were kept in the coastal than in the mainland area (Wald test *t* = 34.225, df = 1, *p* < 0.001). No significant differences in the probability to produce offspring were detected between SM and WM females, adjusting for area (Wald test *t* = 2.053, df = 1, *p* = 0.152) (Figure 6).

**Figure 7:**
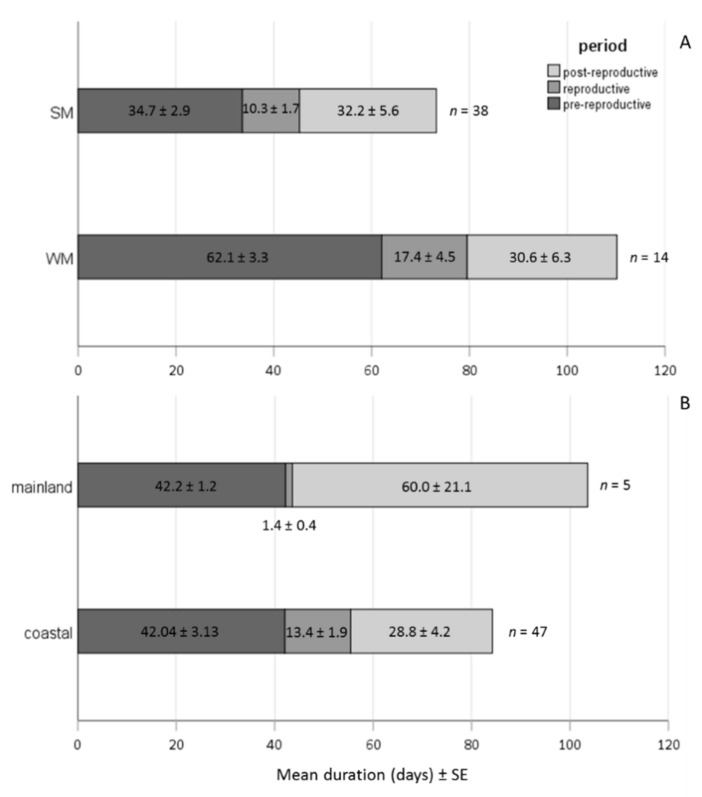
Bars depicting the mean duration of pre-reproductive, reproductive and post-reproductive periods of SM and WM females regardless of the overwintering area (**A**) and of females exposed in the mainland (Naousa) and the coastal (Volos) area, regardless of their phenotype (**B**).

WM females exhibited a significantly longer pre-reproductive period than SM females, adjusting for area (Wald test *t* = 11.179, df = 1, *p* = 0.001). No significant differences were recorded in the reproductive and post-reproductive period (Wald test *t_repr._* = 2.284 *t_post-repr._* = 0.007, df = 1, *p* > 0.05) between SM and WM females (Figure 7A). The offspring production period was longer in the coastal area (Wald test *t* = 6.558, df = 1, *p* = 0.01), adjusting for female phenotype. The post-reproductive period was marginally longer in the mainland than in the coastal area (Wald test *t* = 3.065, df = 1, *p* = 0.08). No significant differences were recorded on the duration of pre-reproductive period of females in the coastal and the mainland area (Wald test *t* = 0.011, df = 1, *p* = 0.917) (Figure 7B).

**Figure 8:**
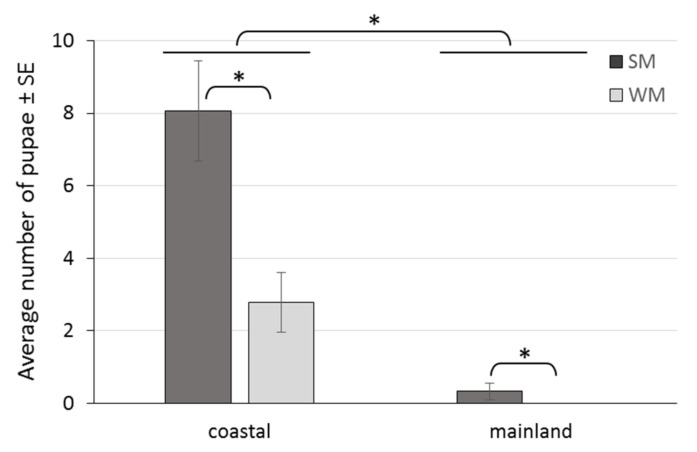
Offspring production by SM and WM females in field conditions of a coastal area (Volos) and a mainland area (Naousa) from 1 November 2019 to 31 May 2020. Values within the chart below the asterisk differ significantly (*p* < 0.001).

The area (coastal, mainland) where females overwintered strongly affected the production of progeny, with 92.3% less pupae recorded in the mainland than in the coastal area (IRR (95% CI) = 0.077 (0.011, 0.515), *p* = 0.008). WM females produced 67.1% less progeny than SM females (IRR (95% CI) = 0.329 (0.169, 0.639), *p* = 0.001), adjusting for area, ambient temperature and days spent in the field (Table 3, Figure 8). Also, SM females produced 38.9 times more progeny in the coastal than in the mainland area (IRR (95% CI) = 38.9 (10.7, 141.0), *p* < 0.001), and 3.03 times more progeny than WM females in the coastal area (IRR (95% CI) = 3.03 (1.56, 5.87), *p* = 0.006) (Table 3, Figure 8).

**Figure 9:**
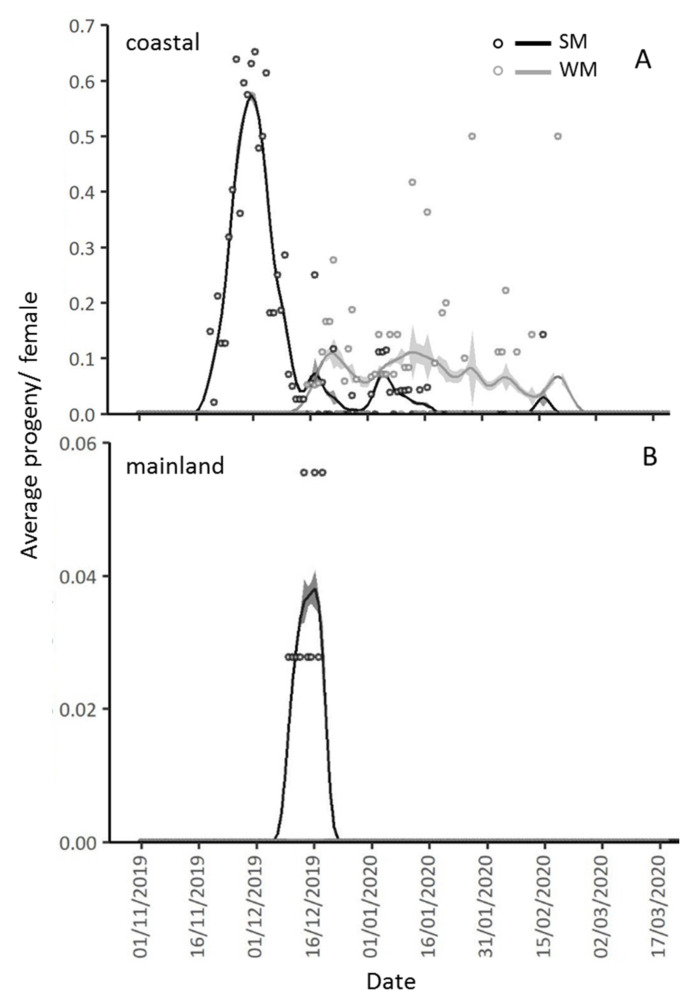
Gaussian Kernel smoothing curves and standard errors (grey zones above and below the smoothing curves) for average daily pupae (circles) per SM and WM female, exposed since 1 November 2019 in the coastal (Volos) and the mainland area (Naousa). In the coastal area, pupae production started 20 days after SM females were transferred to the field and lasted 58 days (from 20 November 2019 to 17 January 2020). Pupae production by WM females in the coastal area started 46 days after their transfer to the field and lasted 67 days (from 16 December 2019 to 21 February 2020) (**A**). In the mainland area, pupae production was recorded 40 days after SM females were transferred to the field and lasted 9 days (from 10 December 2019 until 19 December 2019) while no pupae were produced by WM females in this area (**B**).

In the mainland area, pupae production by SM females was recorded from 10 December 2019 to 19 December 2019, while no pupae were produced by WM females overwintering in the same location. In the coastal area, pupation of offspring from SM females was recorded from 20 November 2019 to 17 January 2020, whereas the first pupa produced by WM females kept in the same location was recorded almost a month later, on 16 December 2019 and the last pupa on 21 February 2020 (Figure 9). The mean daily temperature prevailing in each area did not affect the progeny production, adjusting for female phenotype and days spent in field conditions (IRR (95% CI) = 0.95 (8.98, 1.006), *p* = 0.082) (Table 3). The time that females spent in field conditions significantly affected the number of produced pupae, as for every 10 days spent in the field, progeny production was reduced by 11.5% (IRR (95% CI) = 0.895 (0.817, 0.990), *p* = 0.029). The decrease in pupae production in relation to the time spent in the field was more pronounced in the coastal than in the mainland area (IRR (95% CI) = 0.905 (0.8821, 0.997), *p* = 0.043) (Table 3, Figure 9).

## 4. Discussion

In the present study, we demonstrated that the two phenotypes (summer morphs—SM and winter morphs—WM) of *D. suzukii* adults differ in lifespan and reproduction rates during overwintering in field conditions of a coastal area with a mild winter and a mainland area with a cold winter. Differences in these key life history traits depend greatly on the climatic conditions prevailing in the area of exposure and are more pronounced in colder environments. Our study suggests that this important agricultural pest is characterized by wide thermal tolerance, attributed to the existence of two seasonal phenotypes, confirming its ability to survive and reproduce in a variety of habitats. WM perform better in the cold mainland area where they exhibit a long lifespan and reproductive arrest and SM perform better in the mild-coastal area where they remain reproductively active throughout winter.

Our experiments revealed that WM outlived SM when all individuals were subjected to the same climatic conditions after adult emergence. In addition, WM lifespan was higher in the mainland than in the coastal area. The range of lifespan observed in the current study is in agreement with the findings of Rendon and co-authors [34,41] who recorded the survival of SM and WM kept at 9 °C, 12 °C and 14 °C under constant laboratory conditions. Recent studies have shown that WM exhibit higher cold tolerance than SM and respond better to medium and low temperatures [15,16,34,41], even when SM had been previously acclimated [14]. Physiological, morphological and behavioral adaptations, such as cuticular melanization, accumulation of energetic reserves and cryoprotectants during larval stage, as well as decreased metabolic rates and locomotion in WM adults account for the higher survival rates [28,42]. Gene expression in WM is consistent with cold-hardening mechanisms such as adjustments to ion transport and upregulation of carbohydrate metabolism [12]. Moreover, increased overwintering capability in several Drosophilidae has been positively correlated with high levels of triacylglycerol concentrations [43].

Our results indicate that SM adults were able to overwinter both in mild and in colder winter environments, in contrast to previous research suggesting that SM are not expected to survive long periods at low temperatures and therefore may not be able to overwinter at cold climates [28,44]. The transfer of adults to field conditions early in November, when temperatures fluctuated above 10 °C for almost a month, possibly allowed SM flies to experience acclimatization under gradual-seasonal cold in nature, which later increased their cold tolerance [45]. Increased cold tolerance of SM later in autumn and winter was probably attributed to the accumulation of carbohydrates through nutrition [12,46]. Differences in survival between the two phenotypes were more pronounced in the mainland colder area, indicating that WM perform better at colder conditions than SM. Apart from acclimatization that both phenotypes had undergone during their transfer to field conditions early in autumn, WM had also experienced developmental acclimation at larval and pupal stage during rearing at 15 °C in the laboratory. Physiological changes in gene expression during the development of WM immature stages seem to further promote cold tolerance of emerging adults [47].

Shortly after adult emergence, most SM females had developed ovarioles whereas no WM female was found to be reproductively mature. Additionally, it is known that males of certain Drosophilidae species become sterile after exposure to low temperatures (<12 °C) [48]. During our experimental procedure the reproductive status of newly emerged WM males was assessed indirectly, as males and females of each phenotype were given the opportunity to mate after emergence. Subsequently, even though we did not evaluate the presence of sperm in spermathecae of female SM and WM, offspring production by both phenotypes indicated that matings and sperm transfer had successfully occurred during the 72 h interval before transferring adult flies to field conditions. To our knowledge, this is the first evidence that *D. suzukii* males which develop and emerge at 15 °C and with a short photophase are not sterile and can successfully transfer sperm when given the opportunity.

Additionally, our results showed that, although WM females possess undeveloped ovaries, they engage in copulations and store sperm in their spermathecae before the onset of adverse climatic conditions. Sperm is used for egg fertilization and oviposition later in the season, after they have progressively reached reproductive maturation. As previously proposed, *D. suzukii* overwintering females are able to mate before the arrival of cold temperatures during autumn and lay fertilized eggs the following spring [28,49]. Previous studies have also shown that *D. suzukii* spermathecae are more melanized and/or sclerotized compared to other Drosophilidae, with these adaptations favoring long-term storage protection of sperm from mutagenic ultraviolet-A radiation effects, parasites and dehydration [50]. The strong upregulation of one of the cytochrome P450 (CYP) genes, related with detoxification of xenobiotics and neutralization of possible toxic substances in seminal fluids, has also been characterized as a genetic basis of long-term sperm storage in *D. suzukii* females [50]. Maximizing the viability of sperm obtained before overwintering suggests that *D. suzukii* females may have adapted to the low probability in finding mates during winter.

During our experiments, more than half of the females of both phenotypes produced offspring in the coastal area in autumn and winter, but only a small percentage of SM and no WM females produced progeny in the mainland cold area. Experiencing the mild climate of the coastal area, SM females started ovipositing early in November and remained reproductively active almost throughout winter. The reproductive arrest of WM females was gradually reversed in the coastal area, where after a longer pre-reproductive period than SM females, the onset of offspring production was observed. A different response was recorded in the mainland area where the colder autumn and winter led to a gradual entry of SM to reproductive arrest, with limited offspring production by very few females and to the preservation of reproductive dormancy of WM females that died before reproducing. These two reproductive trends by both *D. suzukii* phenotypes, indicate a strong relationship between the prevailing climatic conditions and the reproductive readiness of *D. suzukii* females, regardless of their phenotype. Unfavorable winter climatic conditions can completely halt or diminish reproduction and favorable conditions can initiate or preserve reproduction of WM and SM, respectively. Taken together, in *Drosophila* spp., the term “reproductive diapause” has been used to describe a general ovarian dormancy, without necessarily requiring that the dormancy would be maintained and terminated by specific cues [33]. Our results are in agreement with previous studies showing that reproductive dormancy of overwintering females in *Drosophila* sp. can be easily induced after exposure to short photophase and low temperatures (<14 h day length, 10–12 °C), and may also impulsively be terminated when conditions become more favorable [30]. On the other hand, brief exposure of reproductively active SM females to a short photophase can significantly decrease the levels of egg maturation and offspring production, even when the temperature is higher than 15 °C [31].

Overwintering females had a shorter lifespan and produced more progeny under the mild winter of the coastal area than when they were exposed to the cold winter of the mainland area. Additionally, SM females lived less and had a higher reproductive output than WM females. These findings indicate that reproductive activity is strongly related to the prevailing climatic conditions and the generated responses to each phenotype and may induce a cost in terms of reduced lifespan to *D. suzukii* females [51,52]. Unfavorable or fluctuating conditions may differently affect the allocation of energetic reserves towards storage than reproduction [34,41]. Short exposure to diapause inducing conditions triggers a hormonal cascade that leads to accessory gland atrophy, pause of yolk accumulation in ovarioles, arrest of mating activity, and several other changes in physiology that stop progeny production and enhance winter survival [31]. The low number of offspring produced by WM females during overwintering in the coastal area may have also resulted from the low fertility of WM males that inseminated them prior to their transfer to field conditions. Besides, it is also known that WM females engaging in copulations after cold exposure have a higher reproductive output later in spring than those mated before winter [14].

## 5. Conclusions

Our findings highlight the successful adaptation of D. suzukii in both areas tested. By manifesting two seasonal phenotypes, this pest can survive winter in the mainland cold area, and can be active throughout winter in the coastal area. Each phenotype confers fitness benefits compared to the other depending on the climatic conditions. Nevertheless, the expression of the two morphs is plastic and can be gradually reversed according to the environmental cues. The data generated in the current paper may provide a solid basis for an area-specific population modelling, depending on the prevailing climatic conditions. Prediction of abundance and infestation potential of the first spring generation of *D. suzukii* has profound practical implications as far as the sound management of this notorious pest is regarded.

## Figures and Tables

**Table 1:** Coordinates of two geographic locations: a coastal area with mild winter in central Greece (Volos) and a mainland area with cold winter in northern Greece (Naousa), where *D. suzukii* adults (SM and WM of males and females) were transferred from autumn 2019 until spring 2020.

Location	Lat. (North)	Long. (East)	Treatment	Number
Coastal: Volos	39°23′17.6′′	22°56′24.6′′	SM females	50
			WM females	25
			SM males	50
			WM males	25
Mainland: Naousa	40°37′45.5′′	22°04′11.0′′	SM females	50
			WM females	25
			SM males	50
			WM males	25

**Table 2:** Comparison of climatic conditions variables using Prais–Winsten regression between a coastal area in central Greece (Volos) and a mainland area in northern Greece (Naousa), from autumn 2019 until spring 2020.

Variable (Ref: Coastal Area)	B (SE)	*p* Value
Maximum Temperature	−3.76 (1.51)	0.013
Mean Temperature	−3.41 (1.61)	0.035
Minimum Temperature	−2.76 (1.52)	0.069
Maximum Relative Humidity	−1.30 (2.42)	0.591
Minimum Relative Humidity	3.91 (3.03)	0.198

**Table 3:** Variables of the generalized estimating equations GEEs with significant effects on the progeny production by *Drosophila suzukii* females in the two areas tested.

Factor	Number of Progeny	
	Wald *t*-Test (df)	*p* Value
Mean Temperature	3.03 (1)	0.082
Area (ref: mainland)	6.99 (1)	0.008
Phenotype (ref: WM)	10.76 (1)	0.001
Days in the field	4.78 (1)	0.029
Mean Temperature × Area	4.09 (1)	0.043
Area × Phenotype	2383.76 (1)	<0.001

## Data Availability

The data presented in this study are openly available in the open public repository Data.World at https://data.world/stelapap/drosophilasuzukiioverwinteringmorphs (accessed on 1 July 2021).
